# Esketamine Use in Treatment-Resistant Depression with Comorbid ADHD and Autistic Traits: A Case Report

**DOI:** 10.1192/j.eurpsy.2026.11092

**Published:** 2026-07-31

**Authors:** E. D. Cindik-Herbrueggen, A. S. Kucukali

**Affiliations:** 1 Neuro-Psychiatrisches Zentrum Riem, Munich, Germany; 2 Psychology, Istanbul Gelisim University, Istanbul, Türkiye

## Abstract

**Introduction:**

Adult attention-deficit/hyperactivity disorder (ADHD) frequently co-occurs with mood and anxiety disorders (Sandstrom et al. Acta Psychiatr Scand 2021;143 380–391), and when major depressive disorder (MDD) is present, it often follows a treatment-resistant course (Thapar et al. J Child Psychol Psychiatry 2023;64 4–15). Intranasal esketamine, a non-competitive NMDA-receptor antagonist, has demonstrated rapid and clinically meaningful benefit in TRD across both short- and long-term studies (Oraee et al. J Psychiatr Res 2024; Reif et al. N Engl J Med 2023;389 1298–1309), but evidence in patients with co-occurring ADHD and autistic traits remains limited.

**Objectives:**

To describe the clinical course, tolerability, and early outcomes of esketamine in an adult with TRD, comorbid ADHD, and autistic traits.

**Methods:**

A 33-year-old man with ADHD and autistic features presented with severe depression and suicidal ideation. History included multiple psychiatric hospitalisations and inadequate response to sertraline, venlafaxine, mirtazapine, bupropion, and melatonin (failures across ≥2 antidepressant classes, consistent with TRD). He also received electroconvulsive therapy (ECT) without sustained benefit. Baseline psychometrics: Beck Depression Inventory-II (BDI-II)=25 (moderate severity); Symptom Checklist-90-Revised (SCL-90-R): Depression=2.38, Obsessiveness=1.90. Intranasal esketamine 56 mg twice weekly was initiated in August 2025 alongside escitalopram 10 mg/day; standard cardiovascular and mental-status monitoring was conducted per protocol.

**Results:**

Treatment was well tolerated. Transient photophobia and mild balance disturbance occurred, but no psychotic symptoms or serious cardiovascular events were observed. Small, self-limited blood-pressure rises were noted. By week 2, the patient reported improved mood, reduced suicidal ideation, and greater daily engagement. Serial psychometrics are ongoing to objectify change.

**Conclusions:**

Esketamine may represent a feasible, rapidly acting treatment option for TRD when ADHD and autistic traits co-exist, with acceptable short-term tolerability after multiple conventional treatment failures. Further case aggregation and prospective studies are warranted to guide integrated, neurodevelopmentally informed management in this population.

**Disclosure of Interest:**

None Declared

